# Popular Instruction

**Published:** 1860-05

**Authors:** 


### POPULAR INSTRUCTION.

Messrs. Editors:—In the Register of November last, appeared an article on the subject of popular instruction in dentistry, deploring the prevailing ignorance among the masses, and suggesting the publication in the newspaper press, of short but plain and attractive essays on the importance of preserving the teeth,—their use in the animal economy, with some general directions as to their preservation, etc.; and in the preparation of which I was careful to enjoin entire freedom from technicalities and "*everything of an advertising*

*character.*" It will be clearly seen, by any one who will turn to the article in question, that there was no endorsement given to "professional advertising" as practiced at this day.

As some indication, though imperfect, of my object and purpose in this matter, I must ask the reader to turn to the January issue of the Register, page 320.

My motive for rehearsing this story is to answer your correspondent, C. C. Dills, who, in an article entitled "professional advertising," and published in your January issue, takes, what I conceive to be, an unwarrantable liberty with the suggestions I have made, in endeavoring, apparently, to make me sustain indiscriminate professional advertising.

Mr. Dills expresses himself as "pleased to note the movement for 'popular instruction'—bearing with it the true principle of dental advertising—that of informing the uninformed of the *importance and capabilities of dentistry.*"

The words which I have italicised express something very different, and a purpose entirely dissimilar from anything I have proposed. My object (and which I think I made plain) was to "inform the uninformed" of the nature and usefulness of their teeth, and the importance of their preservation, with some hints as to the prevention of their decay and loss, while *he* proposes to inform them of "the importance and capabilities of dentistry."

Prominent physicians in our large cities have, on the approach and during the prevalence of an epidemic, brought to the notice of the people, by circular and through the public press, a series of suggestions or hints as to the proper course of conduct or living best calculated to secure exemption from the disease, and designed as a preventive measure solely. Now, the mass of our people require something of the same sort in regard to the decay of their teeth, for the epidemic is raging to an alarming extent, and they should be informed how best to prevent its approach or combat its attack; but this can not be accomplished by informing them of the relative cost of gold and tin fillings, or of the very many methods of supplying the loss of the natural teeth, with prices attached, or of the immense facilities and astounding capacities of the advertiser, which is the customary form of advertising now prevalent, and which I utterly and indignantly repudiate. My object is PREVENTICN; Mr. Dills', I fear, is PRACTICE.

In the sincere hope that I have clearly and satisfactorily explained my position, and what I conceive to be the difference between "popular *instruction*" and "professional *advertising*," I now close, with the single remark that my only purpose in this paper is to keep awake attention to the importance of popular instruction, and but for which, Mr. Dills would have remained unanswered till the "crack of doom," so far as I am concerned. Yours,

PHILADELPHIA, Feb., 1860. O. U. C.

